# Smartphone Usage Among Doctors in the Clinical Setting in Two Culturally Distinct Countries: Cross-sectional Comparative Study

**DOI:** 10.2196/22599

**Published:** 2021-05-10

**Authors:** Anjali Ajay Nair, Samreen Afroz, Bushra Urooj Ahmed, Uzma Urooj Ahmed, Chi Chung Foo, Hind Zaidan, Martin Corbally

**Affiliations:** 1 School of Medicine RCSI Bahrain Busaiteen Bahrain; 2 Department of Surgery, Li Ka Shing Faculty of Medicine University of Hong Kong Hong Kong Hong Kong; 3 Department of General Surgery King Hamad University Hospital Busaiteen Bahrain; 4 Our Lady’s Children’s Hospital, Crumlin Dublin Ireland

**Keywords:** smartphone use, mobile phone, mobile technology, smartphone technology, medical apps, mobile applications, smartphone applications, mHealth, mobile health, digital health, medical informatics, internet, doctors, patient care, point of care, Bahrain, Hong Kong

## Abstract

**Background:**

Smartphones and mobile applications have seen a surge in popularity in recent years, a pattern that has also been reflected in the health care system. Despite increased reliance among clinicians however, limited research has been conducted on the uptake and impact of smartphone usage in medical practice, especially outside the Western world.

**Objective:**

This study aimed to identify the usage of smartphones and medical apps by doctors in the clinical setting in 2 culturally distinct countries: King Hamad University Hospital (KHUH), Bahrain and Queen Mary Hospital (QMH), Hong Kong.

**Methods:**

A cross-sectional, comparative study was conducted where doctors in both hospitals were asked to take part in a 15-item online survey. The questions were categorized into the following groups: demographics of the study population, ownership and main use of smartphones, number and names of medical apps currently owned, rating usage of smartphones for medical purposes, time spent on a smartphone related to clinical use, clinical reliance on smartphones, and views on further integration of smartphones. The results were then tabulated and analyzed using SPSS Statistics 25 for Mac (IBM Corp Inc, Armonk, NY).

**Results:**

A total of 200 doctors were surveyed, with a total of 99.0% (99/100) of the doctors owning a smartphone in both KHUH and QMH; 58% (57/99) and 55% (54/99) of the doctors from KHUH and QMH, respectively, identified communication as their main use of smartphones in the clinical setting (*P*=.004). Doctors from KHUH were likely to spend more time on medical apps than doctors from QMH (*P*=.002). According to the overall results of both hospitals, 48% (32/67) of the junior doctors claimed high reliance on smartphones, whereas only 32.3% (41/127) of the senior doctors said the same (*P*=.03). Of doctors in KHUH and QMH, 78.0% (78/100) and 69.0% (69/100), respectively, either strongly agreed or agreed that smartphones need to be integrated into the clinical setting. In terms of preferences for future apps, 48% (48/100) and 56% (56/100) of the doctors in KHUH and QMH, respectively, agreed that more medical applications need to be created in order to support smartphone use in the clinical setting.

**Conclusions:**

These results suggest a substantial acceptance of smartphones by doctors in the clinical setting. It also elicits the need to establish policies to officially integrate smartphone technology into health care in accordance with ethical guidelines. More emphasis should be placed on creating medical applications that aid health care professionals in attaining their information from accurate sources and also regulate a system to monitor the usage of mobile devices within hospitals to prevent a breach of patient privacy and confidentiality.

## Introduction

### Mobile Health and Smartphones in Health Care

Innovation is one of the hallmarks of medical progress, but traditionally physicians are, as a group, reluctant to embrace all aspects of technical change. As a knowledge-based specialty, the practice of medicine has constantly evolved to ensure wider, faster, and more accurate access to information. Information sharing has changed the way we complete research and share that information for the benefit of our patients. There remains, however, a reluctance for doctors to immerse themselves fully in or on this information highway, and often, this reluctance is based on concerns of confidentiality and a passive acceptance of established practice. Smartphones have had a major impact on rapid access to information and have made major inroads into the mechanisms of information transfer for the profession in general [1]. By and large, smartphones are increasingly used as a means to improve communication between practitioners, keep up to date with the latest medical news and practices, check up on patients post-discharge, look up reference values, and differentials and drug prescriptions as well as provide dynamic education for doctors [1-5]. Studies roughly estimate that 98.4% of doctors own a smartphone and 92% agreed that smartphones positively impacted their practice [2,5-7].

Mobile health (mHealth) is a term used to describe the advances in mobile applications and associated innovations in technology to aid patient care. Being a major constituent of mHealth, mobile devices have drastically evolved since their first introduction in 1973 [1,8]. Doctors are now able to view medical textbooks and calculate drug formulas through apps, eliminating human errors and thus saving time [8,9]. A study on mHealth published in 2017 in Turkey found that communication and information gathering were the major uses of smartphones among physicians [3,10]. With the introduction of smartphones into hospitals, there has also been, peri passu, a simultaneous increase in the demand for medical applications, which has ushered in a new era of convenience. There are currently over 40,000 medical apps available for download in various app stores, and this figure will only continue to rise with increasing popularity [4,11].

### Aim of the Study

Despite this increased dependency, limited research has been conducted on the impact of smartphone usage and how it can change medical practice. While there are some data from the United Kingdom and other Western countries, there remains a lack of research in the Middle East and Asia. In this study, we aimed to identify the usage of smartphones and medical apps by doctors in the clinical setting in 2 culturally distinct countries: King Hamad University Hospital (KHUH), Kingdom of Bahrain and Queen Mary Hospital (QMH), Hong Kong. We attempted to understand how the socioeconomic status and culture of the 2 countries impacts the views of health professionals towards smartphones and their use and acceptance in the clinical setting.

## Methods

### Study Design

We conducted a cross-sectional study over a period of 2 weeks in January 2018 in KHUH, Kingdom of Bahrain and QMH, Hong Kong, both of which are tertiary referral centers and academic hospitals. We designed an online questionnaire on Google Forms based on results from a preliminary literature search [6,7,10]. The questionnaire (which can be found in Multimedia Appendix 1 and Multimedia Appendix 2) consisted of 15 items that were categorized into the following groups: demographics of the study population, ownership and main use of smartphones, number and names of medical apps currently owned, rating usage of smartphones for medical purposes, time spent on a smartphone related to clinical use, clinical reliance on smartphones, and views on further integration of smartphones.

### Study Participants

The questionnaire was open to all doctors irrespective of grade or specialty. To make a fair comparison, we decided to assess the same 15 departments in both hospitals with the responses from the additional departments marked as “others.” The exclusion criteria were medical students, nurses, patients, and hospital administrative staff.

### Data Collection and Statistical Analysis

We set up a booth in an open forum to allow doctors to approach the research investigators. The set-up was kept identical in both hospitals. Participants were given a brief description and purpose of the research, and consent was implied by their agreement to take part in the study. No defining personal information was recorded, and researchers were present to answer any questions raised. Participants completed the questionnaires in person through laptops or iPads provided by the researchers. The questionnaire was administered on Google Forms, and the responses were then downloaded into a Microsoft Excel sheet (Microsoft Excel For Mac Version 16.10.0, Build 18021001, Microsoft Corp, Redmond, WA). We kept the data acquired from the study in an encrypted folder on the principal investigator’s laptop (to remain for 5 years, after which it will be permanently deleted). The results were tabulated and analyzed using SPSS Statistics 25 for Mac (IBM Corp, Armonk, NY).

### Ethics Approval

The study protocol was reviewed and approved by the Institutional Review Board of the University of Hong Kong/Hospital Authority Hong Kong West Cluster (ethics approval number: UW 17-451); KHUH, Bahrain (ethics approval number: KHUH/Research/No. 179/2017); and the Royal College of Surgeons in Ireland Research Ethics Committee (ethics approval number: RCSIBAH15012018).

## Results

The responses from 200 doctors, 100 from each hospital, were recorded, and statistical analysis was performed using SPSS to enable the comparison between the 2 hospitals. The results were tabulated under the headings outlined in the following sections.

### Demographics

A similar demographic pattern was observed, with 24.0% (24/100) of the doctors who participated in the study in each hospital belonging to the age group of 26-30 years, with the same male predominance of 67.0% (67/100) in both the hospitals.

The predominant medical specialty in KHUH was general medicine, with an 18.0% (18/100) response rate, whereas 32.0% (32/100) of the doctors from the department of surgery formed the major specialty who responded in QMH, as shown in Table 1. The demographics of the respondents are shown in Multimedia Appendix 3.

A total of 99.0% (99/100) of the doctors in both KHUH and QMH owned a smartphone. From those who owned a smartphone, 58% (57/99) and 55% (54/99) of the doctors from KHUH and QMH, respectively, identified communication as their main use of smartphones in the clinical setting (*P*=.004; Multimedia Appendix 4; Table 2).

**Table 1:** Medical specialty of the participants in King Hamad University Hospital (KHUH) and Queen Mary Hospital (QMH).

Medical specialty	KHUH (n=100), n	QMH (n=100), n
Accident and emergency	5	1
Anesthetics and intensive care unit (ICU)	14	17
Ear, nose, and throat (ENT)	1	0
General medicine	18	0
Oncology	0	3
General surgery	13	32
Gynecology	5	5
Medicine	0	17
Neurosurgery	1	4
Ophthalmology	3	0
Oral and maxillofacial	1	0
Orthopedic	9	7
Pediatric	11	4
Pathology	0	4
Radiology	5	4
Others	14	2

**Table 2:** Smartphone use by doctors in King Hamad University Hospital (KHUH) and Queen Mary Hospital (QMH).

Main use of smartphones	KHUH (n=99), n	QMH (n=99), n
Search engines	8	23
Camera	1	0
Communication	57	54
Viewing patient information	4	0
Radiology films	0	0
Drug formulas	2	0
Personal use	22	22
All of the above	5	0

### Medical App Ownership

Of the doctors from KHUH and QMH who owned a smartphone, 87% (86/99) and 86% (83/97), respectively, downloaded medical apps; 63% (58/92) of the doctors in KHUH and 51% (48/95) of the doctors in QMH owned 1-3 medical apps, as Table 3 shows.

Medscape and UpToDate were the most used medical apps in KHUH, with 68% (60/88) of the doctors opting for each, while Medscape was the major app used by 68% (57/84) of the doctors in QMH.

**Table 3:** Medical app ownership by doctors in King Hamad University Hospital (KHUH) and Queen Mary Hospital (QMH).

Number of medical apps owned	KHUH (n=92), n	QMH (n=95), n
0	5	11
1-3	58	48
4-5	16	22
≥6	13	14

### Clinical Reliance on Smartphones

The clinical reliance on the apps was analyzed based on different means of attaining information, which involved the following categories: review medical news, hospital information system (online record of patient labs and reports), drug related, communication with patient, communication with colleagues, teaching purposes, training purposes, research purposes, patient education, patient monitoring, and continuing medical education activities.

When asked about the time spent using medical apps in the clinical setting, 29% (29/99) of the total respondents from KHUH used the apps for more than 2 hours per day, 68% (67/99) used apps for 2 or fewer hours, and 3% (3/99) of the doctors never used medical apps, whereas in QMH, 10% (10/97) used apps for more than 2 hours, 83% (80/97) used apps for 2 or fewer hours, and 7% (7/97) never used medical apps in the clinical setting. This is illustrated in Table 4. Doctors from KHUH were likely to spend more time on medical apps in the clinical setting. This difference was significant (*P*=.002, between the 3 time categories).

**Table 4:** Time spent on a smartphone by doctors in King Hamad University Hospital (KHUH) and Queen Mary Hospital (QMH).

Time spent on a smartphone	KHUH (n=99), n	QMH (n=97), n
Never use a smartphone in the clinical setting	3	7
≤2 hours	67	80
>2 hours	29	10

The clinical reliance of doctors on smartphones was recorded on a scale of 1 to 5 (5 being maximum and 1 being minimum). The ratings of 1 and 2 were considered “Low,” 3 was considered as “Neutral,” whereas 4 and 5 were considered “High.”

This was compared with the seniority of the doctors in both the hospitals, where Interns or House Officers and Senior House Officers or Medical Officers were grouped as junior doctors, while Residents, Registrars, or Senior Registrars and Consultants, Associate Professors, or Professors were grouped as senior doctors.

Of the junior doctors in KHUH, 57% (24/42) claimed to be highly reliant on their smartphones in the clinical setting, whereas only 29% (16/55) of the senior doctors said the same. The data at KHUH were significant (*P*=.005).

However, 32% (8/25) of the junior doctors were highly reliant on their medical apps, whereas 35% (25/72) of the senior doctors rated their clinical reliance on smartphones as High in QMH. These data were insignificant (*P*=.81).

According to the overall results in both the hospitals, a significant difference was seen, where 48% (32/67) of the junior doctors claimed high reliance on smartphones, whereas only 32.3% (41/127) of the senior doctors said the same (*P*=.03).

### Results for Future Integration of Smartphones

It was observed that 90.0% (90/100) and 84.0% (84/100) of the doctors in KHUH and QMH, respectively, “Agreed” or “Strongly Agreed” that smartphones have a huge potential in the clinical setting, and 78.0% (78/100) of the participants in KHUH and 69.0% (69/100) of the doctors in QMH “Strongly Agreed” or “Agreed” that smartphones should be formally integrated into the clinical setting.

In addition, 48.0% (48/100; KHUH) and 56.0% (56/100; QMH) agreed that more medical apps need to be created in order to support smartphone use in the clinical setting, and 38.0% (38/100; KHUH) and 55.0% (55/100; QMH) agreed that they would use their smartphones more if there were more medical apps available for use.

### Future Preferences

When asked about the kind of apps they would like to use in the future, drug-related apps formed the major preference for doctors at KHUH (66/100, 66.0%), whereas hospital information systems was the major preference in QMH (65/100, 65.0%), as demonstrated in Table 5.

**Table 5:** Types of future apps preferred by doctors in King Hamad University Hospital (KHUH) and Queen Mary Hospital (QMH). Participants were asked to select up to 3 choices.

Types of preferred future apps	KHUH (n=100), n	QMH (n=100), n
Review medical news	40	44
Hospital information systems	54	65
Drug related	66	64
Communication with patients	24	14
Communication with colleagues	28	31
Teaching purposes	41	17
Training purposes	34	25
Research purposes	38	23
Patient education	0	0
Continuing medical education (CME) activities	37	13
None	3	2
Other	0	0

## Discussion

The study indicates a promising shift in the use of smartphones in the clinical setting. Our study is unique because, to our knowledge, it is one of the only studies that compares the uptake of smartphones of 2 hospitals that come from 2 different cultural backgrounds. It also allows for direct comparison to data collected from Western countries. For example, a study was conducted in the United Kingdom in 2015 where researchers assessed the usage of smartphones among surgeons. Participants from all levels of training were also asked to complete a 13-item questionnaire that assessed their smartphone use in the workplace. Results showed that 93.5% (319/341) owned a smartphone and 54.2% (173/319) within that group also owned medical apps; 79.3% (253/319) stated they would be willing to use their smartphones for hospital-based work [12]. In comparison, our study found higher values of smartphone ownership (99/100, 99.0% in both hospitals) and medical app usage (86/99, 87%, KHUH; 83/97, 86%, QMH), perhaps since the 2 studies were conducted 3 years apart; the later timeframe might have contributed to the differences in values obtained due to the continuous advancements in the field of smartphone technology [5].

Doctors from both countries expressed an interest in the integration of smartphones in the clinical setting, with the majority preferring to see more drug-related apps and hospital information systems in the future. This preference, if accurately employed, could greatly serve to increase efficiency and safety in both drug prescription and accessing patient information, thus saving valuable time.

Compared to previous literature, this study has emphasized a rise in the number of doctors who are clinically reliant on their smartphone. The study, true to its objectives, presents an understanding of the acceptance of technology in the 2 distinct regions.

### Greater Smartphone Reliance Among Junior Doctors Than Senior Doctors

Junior doctors in KHUH were more reliant on smartphones and medical apps than their senior colleagues. However, in QMH, senior doctors were more reliant. This may represent the higher number of senior doctors in QMH (Multimedia Appendix 3).

The results showed that more than half of the junior doctors in KHUH said they were highly reliant on smartphones, whereas the majority of the senior doctors in KHUH asserted low clinical reliance. However, the results in QMH insignificantly varied, with senior doctors being more dependent on medical apps. This could be attributed to the fact that 74% (73/99) of the respondents who participated in QMH were senior doctors, and this could have presented bias in the results.

The results obtained from KHUH are in accordance with a UK-based study done in 2015 within the surgical profession. It was found that junior doctors were more up to date with technology and hence were more clinically reliant on medical apps as compared to their senior colleagues [12]. A similar study conducted by Samsung Medical Center reported that 83.3% of the total access of information via smart devices was also done by junior physicians [13].

With easy and flexible access to medical apps, it has vast potential to enhance current medical practice by attaining fast and comprehensive knowledge, thereby improving the outcome of clinical decisions [4].

### Implications of Smartphone Usage in the Clinical Setting

The majority of participants from both hospitals agreed that smartphones have a future in clinical practice and that doctors would use their smartphones more if there were more apps created. This illustrates a genuine interest from clinicians to optimize smartphone technology in the workplace if (1) smartphones are formally integrated into the clinical setting and (2) more medical apps are created that support smartphone use in medical practice. As a result, the implications can be further categorized based on the different stakeholders involved.

#### Doctors as Users of Smartphones in the Clinical Setting

There is no doubt that smartphones are widely appreciated and used by physicians. Although still emerging in medicine, there have been several reports where doctors have started to officially integrate smartphones into clinical practice. For example, the study conducted at Samsung Medical Center demonstrated how introducing a hospital-specific smartphone app, Dr. SMART S, helped doctors access information regarding inpatients and outpatients (such as lab results) and consultation notes anywhere within the hospital. The app received praise from doctors due to its ease of use and the ability to increase interdepartmental communication [13]. Medical apps have also been slowly introduced in the clinical setting in recent years. In 2015, another study conducted in the United Kingdom assessed how newly qualified doctors used iDoc, an app with medical textbooks that are vital to the doctors’ learning. The results revealed a shift away from hardcopy and electronic textbooks to smartphone textbooks; out of a total of 125 participants, over half said they used the app daily for 12 months. In addition, it was found that the majority of the participants also felt that there was a place for smartphones in the workplace, results that are in line with our research [3].

On a user level, it is also important to consider the factors that can influence the doctor’s experience with and thus their clinical decision making on the smartphone. Special attention should be paid to the design of the app (interface design, app performance, and cost), context within which the interaction occurs, user’s lifestyle, and appropriateness of the activity [14]. Understanding these factors that influence the user experience is an important area of focus for future research of mHealth interventions as they can potentially increase uptake and acceptability of interventions.

#### Monitoring and Regulation of Usage by Hospitals or Governments

There is a huge demand for medical-related apps for health care professionals from many different specialties due to the potential of smartphones to improve efficiency in a variety of aspects in the medical field [15,16]. These advances include, but are not limited to, increased accessibility, portability or mobility, and communicability [2]. In turn, this would help improve the management of patient records, access to online resources, patient monitoring, access to educational aids, and communication among medical staff, all of which contribute significantly to better patient care and hence patient outcomes [13,17,18].

Despite its many possible benefits, smartphone technology also brings to light negative implications that need to be explored before its full integration into health care. One of the major concerns with this type of technology is the potential for a breach of patient confidentiality. To combat this growing problem however, hospitals, where smartphone devices are already employed, have introduced security systems that manage and monitor devices being used within hospital perimeters and also allow for disabling of devices that are not in use anymore [19]. Furthermore, several countries have adopted regulations to step up measures to protect patient information. In the United States, for example, there are several features smartphones must have to comply with the regulations stipulated by the Personal Health Information Protection Act. These include, but are not limited to, the following: encryption of patient information being transmitted along secured networks, password protection of devices, and automatic wiping of data that are no longer required [20]. Similarly, the General Data Privacy Regulation is an initiative that has been established in the European Union that offers strict laws on the management of mHealth that institutions have to adhere to [21].

### Study Limitations

As with the majority of studies, the design of the study is subject to limitations. First, it was predecided — keeping in mind the number of doctors currently in QMH and KHUH — that the study will be concluded after receiving 100 responses in each hospital, with a total of 200 participants. Despite the small sample size, we believe our results are representative of the survey population due to the high response rate and lack of selection bias inherent in an online survey, which also ensured consistency. A possible bias of our study is social desirability bias. The participants, while completing the survey, could have responded in a way that portrayed them in a positive light or would be viewed favorably to us. We tried to minimize this by keeping the participants anonymous so as to remove undue pressure and allow them to respond freely.

Our survey depended on the doctor’s ability to recall past events, which could have introduced recall bias. We took this into account prior to conducting the study by devising a high-quality questionnaire, allowing participants sufficient time for adequate recall, and being present physically at the booth to answer questions they might have [22].

Finally, although participants were selected randomly based on opportunity, it was observed that 73.7% of the doctors recruited in QMH were seniors (Residents, Associate Professors, Professors, or Consultants), making the cohort of senior doctors considerably greater than that of junior doctors. We believe this could be due to the fact they were more willing to participate as they were not as busy as junior doctors. However, as senior doctors form an essential part of the clinical setting in Hong Kong, we chose to include their responses in our study.

### Conclusion

Interestingly, despite cultural differences between Bahrain and Hong Kong, there were no significant differences noted between the results obtained from the 2 countries. These results suggest a substantial acceptance of smartphone technology by doctors in clinical settings. Hence, more emphasis should be placed on creating medical apps that support doctors in patient care, especially for drug-related uses and hospital information systems, which were found to be the major preferences for future apps by doctors in this study.

However, with an increase in the usage of smartphones in the medical field, there are growing concerns about the protection of patient information. There is a need to establish policies to officially integrate the technology in accordance with ethical guidelines and thus encourage future studies to discuss guidelines that are patient-centered and respect patient privacy. This will aid health care professionals in attaining their information from accurate sources and also regulate a system to monitor the usage of mobile devices within hospitals to prevent the breach of patient privacy and confidentiality. Though challenging, implementation of evidence-based guidelines governing smartphone use as well as limiting access to patient information outside of the hospital can help overcome these issues [3,21]. Nevertheless, it is clear through our research and the increased number of collaborative studies on the topic in the last decade [23] that the potential for mobile communication to transform health care and clinical intervention in the community is tremendous [24]. There is, therefore, great scope to harness the potential of mobile use in the clinical setting to improve several aspects of health care [23]. Future studies should aim to expand on the existing research by exploring different contexts, especially ones that compare multiple different contexts such as the current study. This will help to gain a better understanding on whether culture can influence smartphone uptake in the clinical setting.

## Appendix

Multimedia Appendix 1 KHUH questionnaire.

Multimedia Appendix 2 QMH questionnaire.

Multimedia Appendix 3 Table S1: Demographics of participants.

Multimedia Appendix 4 Table S2: Ownership of smartphones and medical apps.

## Abbreviations

KHUH: King Hamad University Hospital
mHealth: mobile health
QMH: Queen Mary Hospital
