# Supplementary material for: Smartphone Usage Among Doctors in the Clinical Setting in Two Culturally Distinct Countries: Cross-sectional Comparative Study
Source: JMIR Mhealth Uhealth. 2021 May 10;9(5):e22599. doi: 10.2196/22599 (PMC8145086; doi:10.2196/22599)
Supplement: Multimedia Appendix 1 [file mhealth_v9i5e22599_app1.pdf]

King Hamad University Hospital, Bahrain

A comparative study to access the usage of smartphones in the clinical setting between different sub-specialties in King Hamad University Hospital (Kingdom of Bahrain) and Queen Mary Hospital (Hong Kong)

* Required

1. **1. What is your gender?** *

   Mark only one oval.

   - Male
   - Female

2. **2. What is your age?** *

   Mark only one oval.

   - 20 to 25
   - 26 to 30
   - 31 to 35
   - 36 to 40
   - 41 to 45
   - 46 to 50
   - 51 to 55
   - 56 to 60
   - 61 to 64
   - 65 or more

3. **3. What is your medical specialty?** *

Mark only one oval.

- Accident and Emergency
- Anaesthetics and ICU
- Ear Nose Throat
- General Medicine
- General Surgery
- Gynaecology
- Neurosurgery
- Oncology
- Ophthalmology
- Oral and Maxilla-Facial Surgery
- Orthopedic
- Pediatrics
- Pathology
- Radiology
- Neonatal surgery
- Other
- Other: ______________________

4. **4. What is your current level of medical training?** *

Mark only one oval.

- Intern
- Senior House Officer
- Registrar
- Senior Registrar
- Consultant
- Other: ______________________

5. **5. How many phones do you own? (If your answer is none, please proceed to question 14)** *

Mark only one oval.

- None
- 1
- 2
- 3
- 4 or more

6. **6. Do you own a smartphone? (If your answer is no, please proceed to question 14)**

Mark only one oval.

- Yes
- No

7. **7. What is the main use of your smartphone? Please choose one:**

Mark only one oval.

- Search engines
- Camera
- Communication
- Viewing patient information
- Radiology films
- Drug formulas
- Personal use
- Other: ____________________

8. **8. Do you have any medical apps? (If your answer is no, please proceed to question 12)**

Mark only one oval.

- Yes
- No

9. **9. How many apps specifically related to medicine do you have on your phone?**

Mark only one oval.

- 0
- 1 to 3
- 4 to 5
- 6 or more

10. **10. Please choose all the medical apps that you currently use:**

Check all that apply.

- [ ] Medscape
- [ ] Figure 1 – Medical Cases for Healthcare
- [ ] UpToDate
- [ ] MayoClinic
- [ ] WebMD
- [ ] Micromedex
- [ ] New England Journal of Medicine
- [ ] Epocrates
- [ ] Read by QxMD
- [ ] Free medical calculators
- [ ] Radiology 2.0
- [ ] Skyscape: RxDrugs and OCM
- [ ] Living Medical Textbooks
- [ ] Medical Radio
- [ ] Neuromind
- [ ] Radiopedia
- [ ] MedShr
- [ ] Twitter
- [ ] Other: ____________________

11. **11. How would you rate your usage of smartphone for medical purposes?**

Mark only one oval per row.

	Never	Low (1 to 2 times per day)	Medium (4 to 5 times per day)	High (6 or more times per day)
Review medical news	◯	◯	◯	◯
Hospital Information Systems (to view electronic health and medical records of patients, view laboratory results, etc)	◯	◯	◯	◯
Drug related (prescription, dosages, contraindications, side effects)	◯	◯	◯	◯
Communication with patients (language translation/diagram)	◯	◯	◯	◯
Communication with colleagues regarding patients (sharing patient info/opinions)	◯	◯	◯	◯
Teaching purposes	◯	◯	◯	◯
Training purposes	◯	◯	◯	◯
Research purposes	◯	◯	◯	◯
Patient education	◯	◯	◯	◯
Patient monitoring	◯	◯	◯	◯
CME activities	◯	◯	◯	◯

12. **12. How long do you spend on your smartphone that is related to clinical use per day?**

Mark only one oval.

- Up to 1 hour
- 1 to 2 hours
- 3 to 4 hours
- 5 to 6 hours
- 6 or more hours
- I do not use my smartphone in the clinical setting

13. **13. On a scale of 1-5 (1 is the least and 5 is the most reliant), how clinically reliant are you on your smartphone?**

Mark only one oval.

	1	2	3	4	5	
Least reliant						Most reliant

14. **14. Please rate the following statements:** *

Mark only one oval per row.

	Strongly disagree	Disagree	Neither agree nor disagree	Agree	Strongly agree
Smart phones have a huge potential in the clinical setting					
Smartphones should be formally integrated more into the clinical setting					
More medical apps need to be created in order to support smartphone use in the clinical setting					
I would use my smartphone more in a clinical setting if there were more medical apps					

15. **15. What kind of apps would you like to see more of? Please choose your top 3:** *

Check all that apply.

- [ ] Review medical news
- [ ] Hospital Information Systems (to view electronic health and medical records of patients, view laboratory results, etc)
- [ ] Drug related (prescription, dosages, contraindications, side effects)
- [ ] Communication with patients (language translation/diagram)
- [ ] Communication with colleagues regarding patients (sharing patient info/opinions)
- [ ] Teaching purposes
- [ ] Training purposes
- [ ] Research purposes
- [ ] Patient education
- [ ] Patient monitoring
- [ ] CME activities
- [ ] None of the above, I would not like to use any medical apps in the clinical setting
- [ ] Other: ____________________

Powered by
Google Forms
