# Supplementary material for: Smartphone Usage Among Doctors in the Clinical Setting in Two Culturally Distinct Countries: Cross-sectional Comparative Study
Source: JMIR Mhealth Uhealth. 2021 May 10;9(5):e22599. doi: 10.2196/22599 (PMC8145086; doi:10.2196/22599)
Supplement: Multimedia Appendix 2 [file mhealth_v9i5e22599_app2.pdf]

Queen Mary Hospital, Hong Kong

A comparative study to access the usage of smartphones in the clinical setting between different sub-specialties in King Hamad University Hospital (Kingdom of Bahrain) and Queen Mary Hospital (Hong Kong)

* Required

1. **1. What is your gender?** *

   Mark only one oval.

   - Male
   - Female

2. **2. What is your age?** *

   Mark only one oval.

   - 20 to 25
   - 26 to 30
   - 31 to 35
   - 36 to 40
   - 41 to 45
   - 46 to 50
   - 51 to 55
   - 56 to 60
   - 61 to 64
   - 65 or more

3. **3. What is your medical specialty?** *

Mark only one oval.

- Accident and Emergency medicine
- Adult Intensive Care Unit
- Anaesthesiology
- Clinical Oncology
- Ear, Nose and Throat
- Medicine
- Neurosurgery
- Obstetrics and Gynaecology
- Opthalmology
- Oral Maxillofacial Surgery and Dental Surgery
- Orthopaedics and Traumatology
- Paediatrics and Adolescent Medicine
- Pathology
- Radiology
- Surgery
- Other: ______________________

4. **4. What is your current level of medical training?** *

Mark only one oval.

- House Officer
- Medical Officer
- Resident
- Professor/Associate Professor/ Consultant
- Other: ______________________

5. **5. How many phones do you own? (If your answer is none, please proceed to question 14)** *

Mark only one oval.

- None
- 1
- 2
- 3
- 4 or more

6. **6. Do you own a smartphone? (If your answer is no, please proceed to question 14)**

Mark only one oval.

- Yes
- No

7. **7. What is the main use of your smartphone? Please choose one:**
Mark only one oval.

- Search engines
- Camera
- Communication
- Viewing patient information
- Radiology films
- Drug formulas
- Personal use
- Other: ______________________

8. **8. Do you have any medical apps? (If your answer is no, please proceed to question 12)**
Mark only one oval.

- Yes
- No

9. **9. How many apps specifically related to medicine do you have on your phone?**
Mark only one oval.

- 0
- 1 to 3
- 4 to 5
- 6 or more

10. **10. Please choose all the medical apps that you currently use:**

Check all that apply.

- [ ] Medscape
- [ ] Figure 1 – Medical Cases for Healthcare
- [ ] UpToDate
- [ ] MayoClinic
- [ ] WebMD
- [ ] Micromedex
- [ ] New England Journal of Medicine
- [ ] Epocrates
- [ ] Read by QxMD
- [ ] Free medical calculators
- [ ] Radiology 2.0
- [ ] Skyscape: RxDrugs and OCM
- [ ] Living Medical Textbooks
- [ ] Medical Radio
- [ ] Neuromind
- [ ] Radiopedia
- [ ] MedShr
- [ ] Twitter
- [ ] Other: ______________________

11. **11. How would you rate your usage of smartphone for medical purposes?**

Mark only one oval per row.

	Never	Low (1 to 2 times per day)	Medium (4 to 5 times per day)	High (6 or more times per day)
Review medical news	◯	◯	◯	◯
Hospital Information Systems (to view electronic health and medical records of patients, view laboratory results, etc)	◯	◯	◯	◯
Drug related (prescription, dosages, contraindications, side effects)	◯	◯	◯	◯
Communication with patients (language translation/diagram)	◯	◯	◯	◯
Communication with colleagues regarding patients (sharing patient info/opinions)	◯	◯	◯	◯
Teaching purposes	◯	◯	◯	◯
Training purposes	◯	◯	◯	◯
Research purposes	◯	◯	◯	◯
Patient education	◯	◯	◯	◯
Patient monitoring	◯	◯	◯	◯
CME activities	◯	◯	◯	◯

12. **12. How long do you spend on your smartphone that is related to clinical use per day?**
Mark only one oval.

- ( ) Up to 1 hour
- ( ) 1 to 2 hours
- ( ) 3 to 4 hours
- ( ) 5 to 6 hours
- ( ) 6 or more hours
- ( ) I do not use my smartphone in the clinical setting

13. **13. On a scale of 1-5 (1 is the least and 5 is the most reliant), how clinically reliant are you on your smartphone?**
Mark only one oval.

	1	2	3	4	5	
Least reliant	( )	( )	( )	( )	( )	Most reliant

14. **14. Please rate the following statements:** *
Mark only one oval per row.

	Strongly disagree	Disagree	Neither agree nor disagree	Agree	Strongly agree
Smart phones have a huge potential in the clinical setting	( )	( )	( )	( )	( )
Smartphones should be formally integrated more into the clinical setting	( )	( )	( )	( )	( )
More medical apps need to be created in order to support smartphone use in the clinical setting	( )	( )	( )	( )	( )
I would use my smartphone more in a clinical setting if there were more medical apps	( )	( )	( )	( )	( )

15. **15. What kind of apps would you like to see more of? Please choose your top 3:** *

Check all that apply.

- [ ] Review medical news
- [ ] Hospital Information Systems (to view electronic health and medical records of patients, view laboratory results, etc)
- [ ] Drug related (prescription, dosages, contraindications, side effects)
- [ ] Communication with patients (language translation/diagram)
- [ ] Communication with colleagues regarding patients (sharing patient info/opinions)
- [ ] Teaching purposes
- [ ] Training purposes
- [ ] Research purposes
- [ ] Patient education
- [ ] Patient monitoring
- [ ] CME activities
- [ ] None of the above, I would not like to use any medical apps in the clinical setting
- [ ] Other: ______________________

Powered by
Google Forms
