# Supplementary material for: Smartphone Usage Among Doctors in the Clinical Setting in Two Culturally Distinct Countries: Cross-sectional Comparative Study
Source: JMIR Mhealth Uhealth. 2021 May 10;9(5):e22599. doi: 10.2196/22599 (PMC8145086; doi:10.2196/22599)
Supplement: Multimedia Appendix 3 [file mhealth_v9i5e22599_app3.docx]

Multimedia Appendix

Table S1: Demographics of participants

|  | | **Hospital** | |
| --- | --- | --- | --- |
|  |  | **KHUH (n)** | **QMH (n)** |
| **Gender** | Male | 67 | 67 |
|  | Female | 33 | 33 |
| **Age** | 20 to 35 | 58 | 60 |
|  | 36 to 55 | 39 | 36 |
|  | 56 to 64 | 3 | 4 |
| **Rank** | Juniors* | 77 | 62 |
|  | Seniors** | 22 | 37 |

*[Juniors- Residents/Registrars/Senior Registrars]

**[Seniors- Consultant/Associate Professor/Professor]
