# Supplementary material for: Smartphone Usage Among Doctors in the Clinical Setting in Two Culturally Distinct Countries: Cross-sectional Comparative Study
Source: JMIR Mhealth Uhealth. 2021 May 10;9(5):e22599. doi: 10.2196/22599 (PMC8145086; doi:10.2196/22599)
Supplement: Multimedia Appendix 4 [file mhealth_v9i5e22599_app4.docx]

Multimedia Appendix

Table S2:Ownership of smartphones and medical apps

|  | | **KHUH**  **n (%)** | **QMH**  **n (%)** |
| --- | --- | --- | --- |
| **Ownership of smartphone**  **(KHUH: n=100, QMH: n=100)** | Yes | 99 (99%) | 99 (99%) |
|  | No | 1 (1%) | 1 (1%) |
| **Main use of smartphone**  **(KHUH: n=99, QMH: n=99)** | Search engines | 8 (8.1%) | 23 (23.2%) |
|  | Camera | 1 (1.0%) | 0 (0%) |
|  | Communication | 57 (57.6%) | 54 (54.5%) |
|  | Viewing patient information | 4 (4.0%) | 0 (0%) |
|  | Radiology films | 0 (0%) | 0 (0%) |
|  | Drug Formula | 2 (2.0%) | 0 (0%) |
|  | Personal Use | 22 (22.2%) | 22 (22.2%) |
|  | All | 5 (5.1%) | 0 (0%) |
| **Medical Apps Ownership**  **(KHUH: n=99, QMH: n=97)** | Yes | 86 (86.86%) | 83 (85.56%) |
|  | No | 13 (13.13%) | 14 (14.43%) |
| **Number of Medical Apps owned**  **(KHUH: n=92, QMH: n=95)** | 0 | 5 (5.43%) | 11 (11.57%) |
|  | 1 to 3 | 58 (63.04%) | 48 (50.52%) |
|  | 4 to 5 | 16 (17.39%) | 22 (23.15%) |
|  | 6 or more | 13 (14.13%) | 14 (14.73%) |
